# Motion Tracking System for Robust Non-Contact Blood Perfusion Sensor

**DOI:** 10.3390/s18010277

**Published:** 2018-01-18

**Authors:** Masaaki Hashimoto, Yoshihiro Taguchi

**Affiliations:** 1 School of Integrated Design Engineering, Keio University, 3-14-1, Hiyoshi, Yokohama 223-8522, Japan; hashimoto@naga.sd.keio.ac.jp; 2 Department of System Design Engineering, Keio University, 3-14-1, Hiyoshi, Yokohama 223-8522, Japan

**Keywords:** blood perfusion, laser Doppler flowmetry, artifact, motion tracking

## Abstract

We propose a motion-robust laser Doppler flowmetry (LDF) system that can be used as a non-contact blood perfusion sensor for medical diagnosis. Endoscopic LDF systems are typically limited in their usefulness in clinical contexts by the need for the natural organs to be immobilized, as serious motion artifacts due to the axial surface displacement can interfere with blood perfusion measurements. In our system, the focusing lens moves to track the motion of the target using a low-frequency reference signal in the optical data, enabling the suppression of these motion artifacts in the axial direction. This paper reports feasibility tests on a prototype of this system using a microfluidic phantom as a measurement target moving in the direction of the optical axis. The frequency spectra detected and the perfusion values calculated from those spectra show that the motion tracking system is capable of suppressing motion artifacts in perfusion readings. We compared the prototype LDF system’s measurements with and without motion feedback, and found that motion tracking improves the fidelity of the perfusion signal by as much as 87%.

## 1. Introduction

A non-invasive and non-contact technique for measuring blood perfusion in the microvascular system is in high demand for clinical diagnosis application. Laser Doppler flowmetry (LDF) measures blood perfusion by detecting backscattered light that is Doppler shifted due to the velocity of the moving red blood cells (RBCs). LDF was first studied in the 1970s [1,2]. Fiber-optic LDF systems can monitor the perfusion change over time in a small volume. However, contact pressure from the fiber probe against the tissue must be minimized during measurements, since microcirculatory perfusion can be significantly altered by external local pressure. Therefore, non-contact sensors are essential to mitigate the uncertainty introduced by external pressure in microvascular blood flow measurement.

Non-contact LDF devices for perfusion monitoring and imaging have recently been developed, and many physiological evaluations—such as a blood perfusion sensing in skin after local heating [3,4], skin burn wounds [5,6], skin tumor [7,8], and mucosa tumor [9,10]—can be performed in a well-controlled environment in which the subject and/or the device were stabilized. Wu et al. assessed small-nerve function by measuring the blood perfusion of a subject’s hand fixed to a stable table [3]. Stucker et al. evaluated the perfusion rates of the different types of skin tumors in patients in a resting posture on a bed to minimize body motion [8]. Sone et al. investigated morphological and physiological changes of vascularity in laryngeal diseases, utilizing a sensor probe fixed to a stabilized desk [9].

From the view point of clinical applications requiring high accuracy and ease of use, motion artifacts due to state changes caused by interference in the Doppler-shifted signal need to be eliminated. These motions cause fluctuations in the perfusion measurements which compromise the interpretation of the data for the sake of diagnosis. These motion artifacts are difficult to separate from time-series perfusion data [11]. Several experimental and analytical approaches have been proposed for overcoming misinterpretations introduced by motion artifacts [12,13,14,15]. Conventional non-contact LDF techniques are often impractical for clinical use, however, because of limitations on the spatial and temporal resolution of conventional techniques.

Especially with endoscopic LDF measurement of the digestive system, natural organ motility introduces strong motion artifacts into perfusion signals that are difficult to remove in data processing steps [16,17]. Motion arises from contraction and dilatation of internal organs—such as gastric motility, beating of the heart, and breathing—where the surface displacement in the axial direction of contraction and dilatation is dominant. Gastric contraction motion is observed during the endoscopy [18], and quantified by captured MRI images [19]. As a basic sensing method for a clinical endoscopic perfusion measurement, therefore, we proposed the non-contact LDF measurement system with focus-based motion tracking that is highly robust to the motion artifacts due to the axial surface displacement. In the proposed system, the focusing lens is controlled to maintain the optical working distance against the motion by axial surface displacement in the LDF perfusion measurement. To compensate for the motion artifacts due to the lateral displacement in the direction of *x*- and *y*-axis or pitch/yaw, the image based calibration techniques, such as the lateral tracking system with a scanning ophthalmoscope demonstrated by Christy et al. [20], could be utilized to the present system by integrating the two-axis mirror scanner.

Various displacement detection methods for auto-focusing have been widely proposed in many fields, such as the image sharpness method for digital cameras [21], the astigmatic method for DVD pickup head [22], the eccentric light beam method for the microscopy [23], and the pattern projection method for the photolithographic process [24]. The frequency filtering approach based on the confocal optical configuration was utilized in our focal control method for the reduction of the motion artifacts, which is highly compatible to LDF measurement setup. The optical reference signal for the feedback control is detected under the confocal configuration, followed by the low-pass filtering processing, since low frequency signal is dominantly dependent on the axial surface displacement. Simultaneously Doppler-shifted signal from the moving RBCs is detected by the same optical path, with high pass filtering processing.

In this study, the feasibility study of our proposed system for the suppression of the motion artifacts in the axial direction prior to the in vivo/clinical tests was conducted using the phantom-based testing.

## 2. Principle

**Figure 1:**
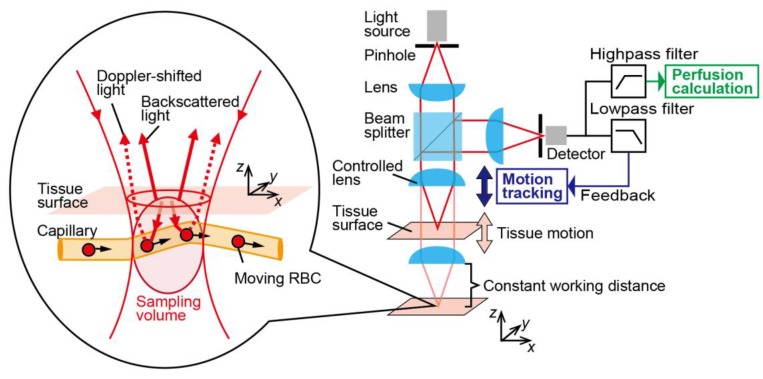
Principle of motion tracking laser Doppler flowmetry system. Doppler-shifted signal is detected from interference between the light backscattered from the surface tissue and light backscattered from moving RBCs. Lens is controlled to maintain a constant distance to the tissue surface using feedback from the signal with a low-pass filter applied, and perfusion is calculated from the high-pass filtered signal.

Blood perfusion, which is defined as the blood volume flow through a mass of tissue (in unit of mL/100 g/min), represents blood flow in the microvascular system. Figure 1 shows the schematic of the principle of the system. In our method, a Doppler-shifted signal is detected from interference between the light backscattered from the surface tissue and light backscattered from moving RBCs within the small sampling volume determined by the optical properties of the confocal optical configuration. In general, confocal optical configurations have high 3D spatial resolving power [25], enabling the optical spatial sectioning of blood vessels and/or a few capillaries. The signal intensity of the power spectra in the frequency domain depends on the velocity distribution of the RBCs within the sampling volume. As the flow velocity increased, the perfusion spectra broadened in the high-frequency region. The first moment of the power spectra is linearly proportional to the root mean square velocity of moving RBCs, so the relative blood perfusion value can be written as [26]

(1) $$\mathrm{Perfusion}\;\mathrm{value}\;\propto \int_{\omega_{0}}^{\omega_{1}}\omega \cdot P\left(\omega \right)d\omega$$

where *P*(*ω*) is the power spectra density of the perfusion signal in the frequency region. *ω*_0_ and *ω*_1_ are the lower and upper limits of the frequency measurements, respectively. A motion artifact is introduced when fluctuating reflected light from the moving tissue surface is combined with the Doppler signal. The power spectra *P*(*ω*) in frequency domain is strongly affected by the intensity of the light reflected from the surface.

Karlsson et al. found that the intensity of surface reflection has a large influence on the perfusion signal when the tissue surface is moving [13]. Especially when measuring the mucosa of inherently motile organs with an endoscope, strong reflection from the mucus causes a strong motion artifact. This is one reason for the difficulty in achieving robust blood perfusion sensing with instruments that cannot be stabilized in the clinical context, such as in an endoscopic environment.

To suppress motion artifacts during blood perfusion sensing, motion-tracking systems have implemented in LDF systems to control the configuration of confocal optical elements. The distance from the sample to the optical components is kept constant by a lens actuator responding to a feedback signal, resulting in suppressing the fluctuations in intensity caused by motion of the tissue surface. Therefore, perfusion measurements taken with this system are robust to the axial motion by tracking the sample at a fixed working distance as shown in Figure 1. Confocal optical configurations have high sensitivity to the surface axial displacement [27,28,29], so the total intensity of the light depends on the distance from the lens to the target surface. The displacement signal for motion tracking and the Doppler-shifted signal are obtained simultaneously by a single detector. For the feedback control system, the low frequency component of the reflected light is exploited as a reference signal. This reference signal from the relative displacement between the lens and surface can be separated from the perfusion signal with signal filtering based on the difference in the target frequencies. The lower frequency limit in Equation (1) is determined by the cutoff frequency of the high-pass filter.

## 3. Experimental Setup

### 3.1. Microfluidic Phantom

**Figure 2:**
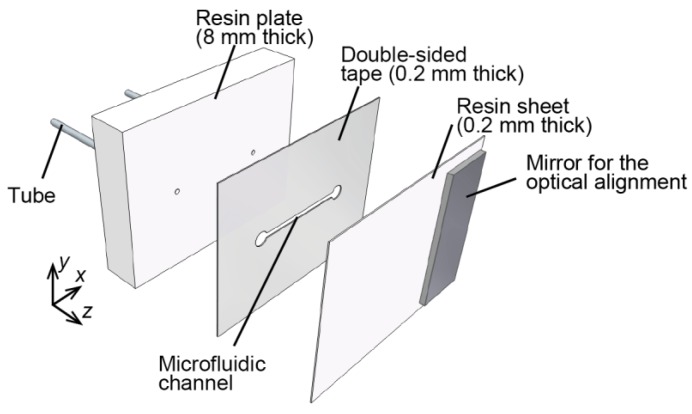
Components of the microfluidic phantom. A 0.2-mm-depth flow channel is positioned at 0.2 mm deep. Flow channel was formed by cutting a piece of double-sided tape, and 0.2-mm-thick resin sheet was adhered to the resin plate with the double-sided adhesive tape.

A microfluidic phantom with an automatic staging platform was adopted as a model for moving vascular tissue. To evaluate the axial motion artifacts due to the change of interference state caused by fluctuating the surface reflected light, the thin-film microfluidic phantom which had a single microchannel parallel to the surface was fabricated using a simple fabrication technique compared with other complicated micro fabrication technique such as poly (dimethylsiloxane) (PDMS)-based technique. The phantom consisted of a diffuse scattering resin (Polyoxymethylene: POM), surrounding a microchannel cut with a laser into double-sided adhesive tape. As shown in Table 1, POM has the absorption coefficient and reduced scattering coefficient which are similar to those of real tissue [30]. Figure 2 diagrams the microfluidic phantom sample. The flow channel was formed by cutting a piece of double-sided tape (MGCS20, Kyodo Giken Chemical, Tokorozawa, Japan) with a laser cutter (MERCURY, GCC, Taiwan). A 0.2-mm-thick resin sheet was adhered to the resin plate with the double-sided adhesive tape. The microfluidic channel 1 mm in width and 0.2 mm in depth, and polymer tubes (1538, IDEX, Lake forest, IL, USA) were connected through the resin plate to supply a RBC solution. A mirror for the alignment of the confocal optics was bonded onto the surface.

**Figure 3:**
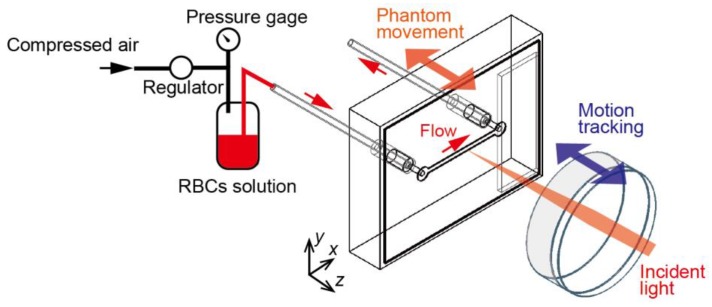
Blood flow in the microfluidic channel and phantom platform movement in the direction of the optical axis. The flow velocity of the RBCs solution in the channel can be controlled by adjusting the pressure. The concentration of the RBC solution (derived from cow hemoglobin) is 20 mg/mL.

The RBC solution was injected into the microchannel at a determined flow velocity, as shown in Figure 3. The pressure of the compressed air driving the flow was measured by the pressure gauge. The flow velocity of the RBCs solution in the channel can be controlled from 0 mm/s to 31 mm/s by adjusting the pressure. The flow rate was estimated from the volume of the solution emitted from the flow outlet. The RBC solution was prepared by dissolving dried cow hemoglobin (HG3760-100G, Sigma-Aldrich, St. Louis, MO, USA) in pure water at a max dissolvable concentration of 20 mg/mL, which is lower than real blood. This is because of prevention of the concentration change due to the sedimentation of RBCs.

The motion of the phantom was controlled by an automatic staging platform to follow a sinusoidal trajectory (frequency: 0.1 Hz, amplitude: 1 mm) in the direction of the optical axis. The motion is variable to the region of internal organs. At the esophagus, for example, fast motion which arises from the beating heart is observed. In the experiment, gastric motility, which is a smooth periodic motion (about three times per minute in the stomach [31], and about seven times per minute in the small intestine [32]) was simulated depending on the response speed of the system.

### 3.2. Motion-Tracking LDF System

**Figure 4:**
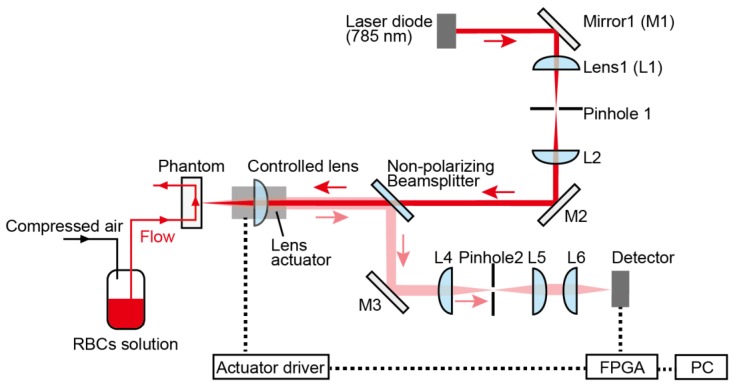
Confocal optical configuration of motion tracking LDF system. A 785-nm-wavelength probe beam is emitted from the diode laser. The controlled lens is mounted on the lens actuator, and is driven by the feedback signal from field-programmable gate array (FPGA).

To verify the functionality of our proposed motion-tracking LDF system, we constructed a prototype system, using the microfluidic phantom as a measuring target. Figure 4 shows a schematic of the experimental apparatus we used.

The probe beam is emitted from a laser diode (L785P090, Thorlabs, Newton, NJ, USA; 785 nm) the temperature of which was kept at 23 °C. The beam is collimated by the lens and focused on the phantom with an achromatic doublet lens (AC254-030-B, Thorlabs, USA) mounted on a lens actuator. A shaft motor (S120T, Nippon Pulse Motor, Tokyo, Japan) works as the lens actuator, and is driven by the feedback signal from a driver (MADHT1105L01, Panasonic, Kadoma, Japan). The scattered and reflected light from the phantom passes through this actuated lens, and is guided by a non-polarizing beam splitter to the detector (2001-FS-M, Newport, Irvine, CA, USA). The reflected signal from the phantom is focused through a pinhole (P75S, Thorlabs, USA; Diameter: 75 μm) onto the detector surface. These optical elements must be strictly aligned using the light reflected from the mirror attached to the phantom surface.

**Figure 5:**
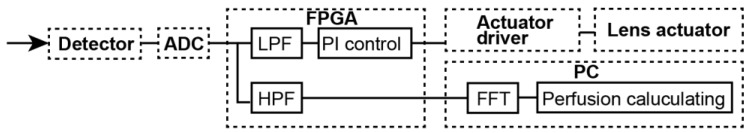
Signal processing flow for motion-tracking and perfusion calculations. The analog signal (sampling rate: $1\times 10^{5}$ samples/second) is low-pass-filtered (20 Hz, 3 dB) for feedback control and high-pass-filtered (1 kHz, 3 dB) for perfusion measurements.

Signal processing for the motion tracking module was based on the high-speed field-programmable gate array (FPGA) module in LabVIEW™ (National Instruments Inc., Austin, TX, USA). Figure 5 shows a flow chart of the signal processing steps. The embedded controller (sbRIO-9637, National Instruments, Austin, TX, USA) with an integrated A/D converter, FPGA, and digital I/O was used for the feedback signal calculation. The analog signal from the detector was DC-coupled, digitalized at A/D converter (sampling rate: 1 × 10^5^ samples/second), and lowpass-filtered to extract the reference signal that communicates the motion of the tissue surface. The cutoff frequency is set to the frequency at several tens of hertz (in this case 20 Hz, 3 dB), considering the phase response of LPF. The FPGA-based proportional integral (PI) control was executed to track the motion of the phantom. In the experiment, the target value for PI control is set to the intensity where focal point is positioned to the center of the channel. The position of the channel was estimated by the optical profile of the depth-scanned data. When the motion tracking is switched ON, the command output value is calculated from the deviation between the low-pass filtered reference signal and the target value.

During signal processing, the frequency analysis for the perfusion calculations is conducted simultaneously with the motion-tracking feedback calculations. The analog signal (sampling rate: 1 × 10^5^ samples/second) is high-pass-filtered (1 kHz, 3 dB) to distinguish between the perfusion signal and the displacement information in the time domain. The perfusion spectra are obtained with fast Fourier transforms of the beat signal in time-domain, calculated from 1024 points of the sampled AC signal. The perfusion spectra *P*[*ω*] used for the perfusion calculations are obtained by averaging the spectra 10 times. The relative blood perfusion signal was calculated as [26]

(2) $$\mathrm{Perfusion}\;\mathrm{signal}\;=\;\overset{F_{2}}{\underset{\omega =F_{1}}{\sum }}\omega \cdot P\left[\omega \right]$$

where *P*[*ω*] is the estimated spectral density of the perfusion signal, and *F*_1_ and *F*_2_ are set to 1 kHz and 39 kHz, respectively. One perfusion signal is calculated approximately every 0.1 s. The perfusion data is gathered in real time, and the FFT spectra and blood perfusion values are calculated and displayed simultaneously.

## 4. Results and Discussion

### 4.1. Transient Perfusion Spectra Variability with and without Tracking

**Figure 6:**
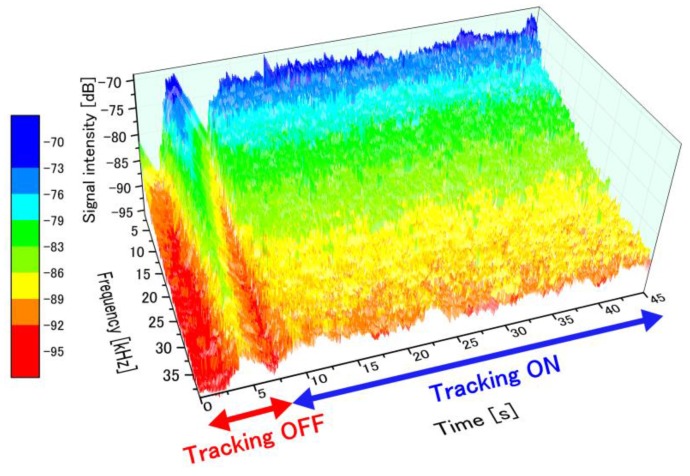
Time-dependent perfusion spectra during the entire measurement period (45 s). Optical tracking was switched ON from the OFF state at 7.5 s, and the optical tracking began at 9 s, after a transient response. The flow velocity was constant at 27 mm/s over this whole period.

**Figure 7:**
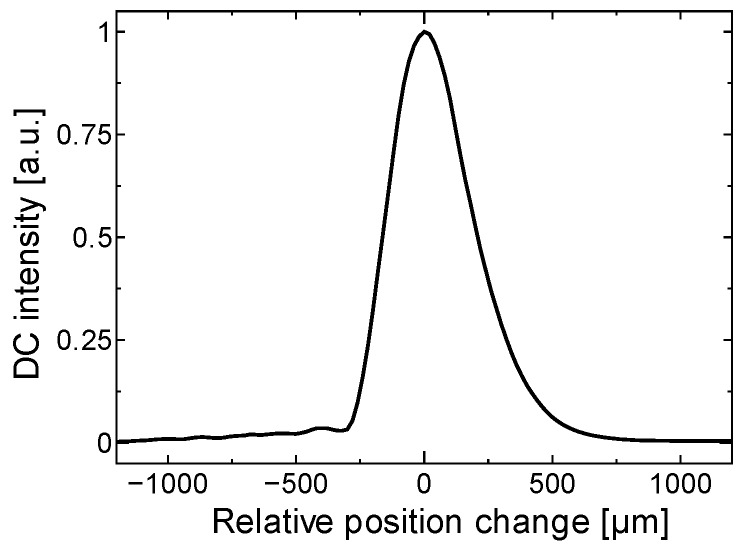
The fluctuation of DC signal intensity with measurement surface displacement. With tracking OFF, the change in DC signal influences perfusion spectra through the heterodyne effect.

To examine the suppression of the fluctuations in the perfusion spectra introduced by the motion tracking feedback, stability of the detected FFT signal was measured with and without motion tracking. Figure 6 presents the time-dependent perfusion spectra over the entire measurement period. The flow velocity in the microfluidic channel of the phantom was maintained at 27 mm/s during the entire period. Motion tracking was switched ON from the OFF state at t = 7.5 s. After a transient response, the controlled lens began to track the motion of the phantom surface (t ≥ 9 s). The perfusion spectra fluctuated significantly without motion tracking due to the appearance of motion artifacts. On the other hand, highly-stable perfusion spectra were captured with motion tracking turned on. Figure 7 shows the light profile axially reflected into our optical configuration, and the fluctuation in accordance with this optical profile directly influenced the spectra measurements.

**Figure 8:**
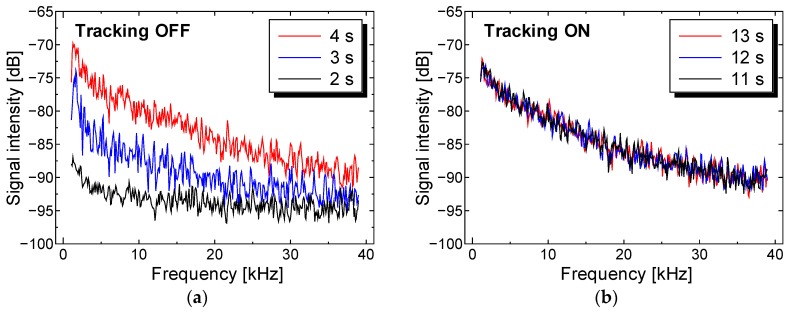
Stabilization of perfusion spectra with motion tracking. Flow velocity was constant at 27 mm/s. (**a**) Perfusion spectra with tracking OFF for 2, 3 and 4 s; (**b**) with tracking ON for 11, 12, and 13 s.

The perfusion spectra *P*[*ω*] during each period with and without motion tracking are shown in Figure 8a,b, respectively. The heterodyne effect strongly affected the gradient and the baseline of the perfusion spectra, so it is difficult to distinguish between the motion artifact and the change in perfusion value. If we focus on the intensity of perfusion spectra at 10 kHz frequency during the entire measurement, the perfusion spectra with tracking were stabilized within a range from a maximum −78 dB to a minimum −83 dB, while the perfusion spectra without tracking fluctuate within a range from a maximum −78 dB to a minimum −92 dB.

### 4.2. Temporal Perfusion Spectra with Motion Tracking

**Figure 9:**
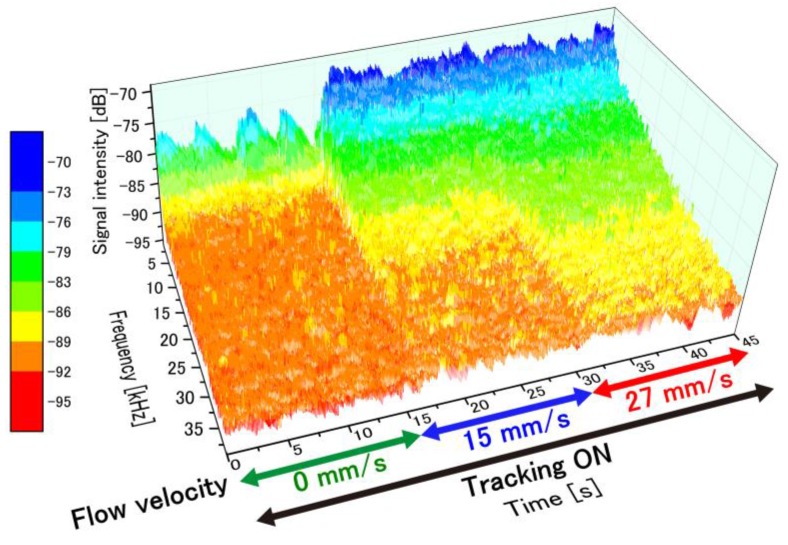
Perfusion spectra changing in time during the entire measurement period (45 s). Flow velocity was controlled at three different flow velocities (0 mm/s from 0–15 s, 15 mm/s from 15–30 s, and 27 mm/s from 30–45 s).

The temporal variation in the perfusion spectra under feedback control was examined by varying the flow velocity. Figure 9 presents a plot of the time-dependent perfusion spectra measured from the moving test platform with motion-tracking engaged. The flow velocity in the microchannel was varied by adjusting the air-pressure regulator (0 mm/s from 0–15 s, 15 mm/s from 15–30 s and 27 mm/s from 30–45 s). The temporal variations in the perfusion spectra caused by these changes to the flow velocity are clearly observed with motion tracking engaged. As the flow velocity in the phantom increased, the perfusion spectra broadened in the high-frequency region as expected. At 0 mm/s, a relatively significant fluctuation in the low frequency region was observed since speckle noise from the surface appeared in the weak signal. This noise source has a negligibly small effect in a signal with a high signal–noise ratio. 

**Figure 10:**
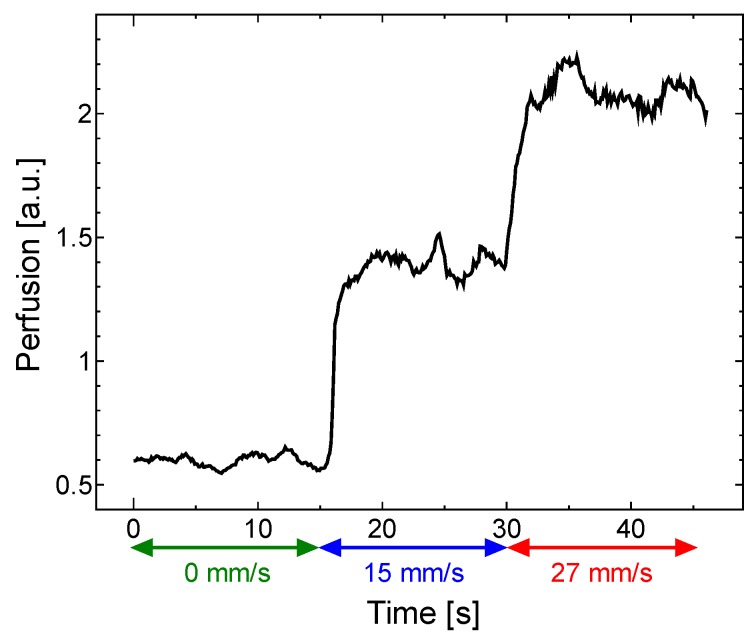
Time-dependent perfusion signals calculated from perfusion spectra during the entire measurement. Perfusion signal increased rapidly when flow velocity increased.

Figure 10 shows the perfusion values over time calculated from the perfusion spectra in Figure 9 using Equation (2). The perfusion values were calculated in 0.1 ms increments. Note that the perfusion value increased rapidly when the flow velocity increased.

**Figure 11:**
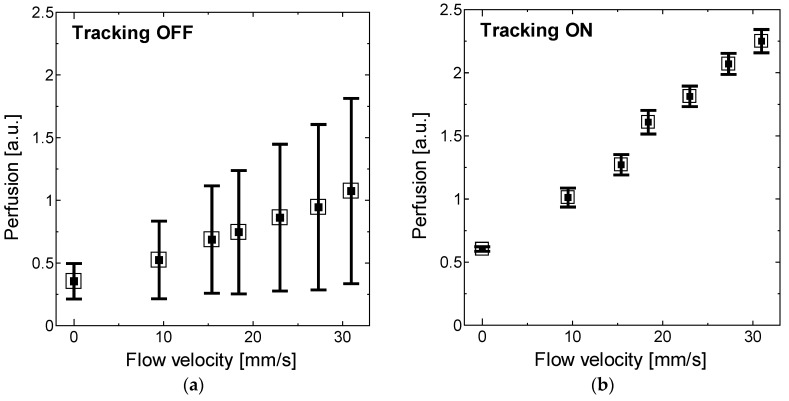
Perfusion value (mean and standard deviation over 20 s) for different flow velocities ranging from 0 to 31 mm/s (**a**) without tracking engaged; and (**b**) with tracking. The standard deviation is correlated with motion artifacts.

Finally, the perfusion values measured from seven different flow velocities with and without motion tracking were compared as shown in Figure 11a,b. The perfusion values without feedback control engaged were extremely scattered and underestimated due to the heterodyne effect on the DC signal. To quantify the degree to which motion artifacts are suppressed, a reduction parameter R was defined as a ratio of the difference in standard deviation with and without motion tracking to the standard deviation without motion tracking (SD_OFF_-SD_ON_/SD_OFF_). R indicates a maximum of 87% reduction in standard deviation of the readings at 31 mm/s and a minimum 75% reduction in noise at 9.5 mm/s. These reductions to the motion artifacts confirm that the motion-tracking feedback system allows for effective LDF measurements without stabilizing the tissue surface in the direction of the optical axis.

## 5. Conclusions

For non-contact LDF measurements, motion artifacts present a serious challenge for clinical endoscopic applications. We developed a prototype motion-tracking laser Doppler flowmetry system that eliminates motion artifacts in the axial direction by controlling the lens positon with a feedback signal representing tissue motion. To demonstrate the feasibility of the proposed technique, a prototype motion tracking LDF system was constructed, and a flow phantom platform was used as the measurement target. The detected perfusion spectra were stabilized with motion tracking, and motion artifacts were reduced by a maximum of 87%. This confirms the feasibility of the proposed sensing system. For future work, we are working on the development of the blood perfusion imaging system which simultaneously compensates the axial/lateral displacement, and the miniaturization of the system for endoscopic applications.

## Figures and Tables

**Table 1:** Optical properties of POM at wavelength of 785 nm.

Optical Property	Value
Refractive index: *n*	1.4 [33]
Absorption coefficient: $\mu_{a}$ [1/cm]	0.03 [34]
Reduced scattering coefficient: $\;{\mu^{\prime }}_{s}$ [1/cm]	20 [34]
